# Charles Bonnet syndrome: taking another look at visual hallucinations in sight loss

**DOI:** 10.1177/25158414251359588

**Published:** 2025-07-29

**Authors:** Lee Jones, Mariya Moosajee

**Affiliations:** Institute of Ophthalmology, University College London, 11-43 Bath St, London EC1V 9EL, UK; Institute of Ophthalmology, University College London, London, UK; Moorfields Eye Hospital NHS Foundation Trust, London, UK; The Francis Crick Institute, London, UK

**Keywords:** Charles Bonnet syndrome, Visual hallucinations

Charles Bonnet syndrome (CBS) manifests as vivid, silent visual hallucinations in people with visual impairment, ranging from brief flickers of colourful geometric patterns to elaborate tableaux of faces, animals and fantastical landscapes. Once little known outside specialist circles, awareness of CBS has grown significantly in recent years. There is now broader recognition that these hallucinations are not indicative of psychiatric illness, but rather reflect the brain’s remarkable propensity to generate visual experiences when sensory input is diminished.

This special collection showcases the multidisciplinary nature of CBS research, bringing together insights from optometry, ophthalmology, cognitive neuroscience and psychology. The studies explore a range of priority areas, including prevalence, underlying neurocognitive mechanisms, psychosocial impact and approaches to clinical management. The publication of this collection reflects growing momentum in CBS scholarship and a broader commitment to better understanding, and more effectively responding to, the complexities of this historically under-recognised condition.

Prevalence was examined in a prospective cross-sectional study of Danish patients with geographic atrophy (GA), revealing a CBS rate of 20.8%, approximately one in five individuals.^1^ This supports earlier findings linking CBS to central vision loss and macular pathology.^2^ The authors call for routine CBS assessment in GA trials and standard ophthalmic care, advocating for sensitive, patient-friendly language when broaching the topic (e.g., ‘Some people with similar eye problems report seeing things that aren’t really there. Have you ever experienced this?’). This aligns with recent recommendations promoting open, empathetic conversations about CBS in clinical settings.^3^

The underlying mechanisms and pathophysiology of CBS were investigated in a study examining neurochemical changes associated with visual hallucinations. A prominent theory suggests that CBS results from an imbalance between excitatory and inhibitory activity within the visual system.^4^ To test this hypothesis, proton magnetic resonance spectroscopy was used to measure levels of glutamate (excitatory) and GABA (inhibitory) in the visual cortices of six CBS patients and matched controls.^5^ No significant group differences were observed, providing no support for the excitation/inhibition imbalance or deafferentation hypotheses. These findings underscore the need for further research using dynamic imaging techniques to capture neural activity during hallucinatory episodes.

A detailed review draws parallels between CBS and other perceptual phenomena such as phantom limb syndrome and tinnitus, all of which can emerge following sensory loss.^6^ The review bridges a conceptual gap by identifying shared cortical, emotional and cognitive factors that may contribute to the onset and persistence of ‘phantom’ experiences. The authors propose an integrated, transdiagnostic framework for understanding these conditions and suggest that leveraging cross-condition insights could accelerate discovery and intervention development. Such integration may enhance understanding of how the brain adapts to sensory deprivation and support the identification of novel therapeutic strategies.

The psychosocial dimensions of CBS were examined in several studies. One study explored the relationship between emotional well-being and individuals’ appraisal of their hallucinations.^7^ Negative affect (i.e., feelings of dysphoria, anger, contempt) and loneliness were significantly associated with a greater perceived impact, with participants who reported higher levels of negative emotion more likely to find their hallucinations distressing and isolating. These findings highlight the importance of addressing emotional well-being as part of the clinical response to CBS.

A second study focused on younger adults (aged 18–60), examining associations between anxiety, depression and hallucination experience.^8^ Anxiety was linked to increased hallucination frequency, though not with duration or emotional effect. The direction of this relationship remains unclear: whether anxiety increases vulnerability to hallucinations or whether hallucinations exacerbate anxiety.^9^ The study reinforces emerging evidence that CBS is not exclusive to older adults,^10^ pointing to the need for age-inclusive diagnostic frameworks and psychological support.

Further insights into lived experience came from a qualitative study with visually impaired military veterans.^11^ Although many participants described their hallucinations as non-threatening, their frequency, disruption to daily activities and uncertain origin were often sources of distress. Notably, limited engagement or discussion with healthcare professionals about CBS was commonly reported, signalling a persistent gap in routine clinical care.

Service delivery was evaluated in a study assessing CBS management before and after the COVID-19 pandemic in a UK hospital eye service.^12^ Patient records revealed inconsistent documentation and limited formal recognition of CBS. Medical retina clinics saw the highest caseloads, consistent with findings on CBS prevalence in macular disease.^1^ The authors encourage routine screening in high-burden subspecialties to support more efficient, risk-informed case identification and better resource allocation.

Collectively, these studies advance both academic and clinical understanding of CBS and emphasise its relevance across disciplines. Despite being a common consequence of visual impairment, CBS remains under-researched and inconsistently addressed in practice. As interest in CBS grows, it is essential that future research targets global evidence gaps and that sustained investment is made in high-quality studies. It is hoped that this collection will stimulate ongoing inquiry and collaboration, ultimately contributing to more effective, evidence-based care for individuals living with CBS.

## Declarations

**Ethics approval and consent to participate:** Not applicable.

**Consent for publication:** Not applicable.

**Availability of data and materials:** Not applicable.
